# Supplementary material for: Benchmark of tools for in silico prediction of MHC class I and class II genotypes from NGS data
Source: BMC Genomics. 2023 May 9;24:247. doi: 10.1186/s12864-023-09351-z (PMC10170851; doi:10.1186/s12864-023-09351-z)
Supplement: Supplementary file 1 — Supplementary Material 1 [file 12864_2023_9351_MOESM1_ESM.docx]

|  | (Freely) available for academic use | FASTQ or BAM input files from WGS, WES and/or RNA-Seq | Running on  Ubuntu 20.04 |
| --- | --- | --- | --- |
| ALPHLARD(-NT) | ✗ |  |  |
| ATHLATES |  |  | ✗ |
| HLAProfiler |  |  | ✗ |
| HLAreporter |  |  | ✗ |
| HLAssign |  | ✗ | ✗^*^ |
| OncoHLA | ✗ |  |  |
| PolyPheMe | ✗ |  |  |
| SNP2HLA |  | ✗ |  |
| SOAP-HLA |  |  | ✗ |

* Latest version of HLAssign is a Windows GUI tool

**Table S1**. Overview of tools that were not benchmarked in our study and the reason for their exclusion. Excluded tools were either not *freely available for academic use*, do not use *FASTQ or BAM input files from WGS, WES and/or RNA-Seq* experiments (e.g., enrichment of the HLA region prior to sequencing is needed) or we were not able to get them *running on Ubuntu 20.04*.
