# Supplementary material for: Benchmark of tools for in silico prediction of MHC class I and class II genotypes from NGS data
Source: BMC Genomics. 2023 May 9;24:247. doi: 10.1186/s12864-023-09351-z (PMC10170851; doi:10.1186/s12864-023-09351-z)
Supplement: Supplementary file 2 — Supplementary Material 2 [file 12864_2023_9351_MOESM2_ESM.docx]

|  | Alignment method | Score function | | | Tool-specific steps |
| --- | --- | --- | --- | --- | --- |
|  |  | **Jointly optimized for allele pair** | **PHRED score used** | **Prior population frequencies** |  |
| HLA*LA | BWA-MEM | yes | yes | no | Graph-based optimization of alignment |
| Kourami | BWA-MEM | yes | yes | no | Graph-guided assembly (post-alignment) |
| arcasHLA | Kallisto  (pseudo-alignment) | hybrid | no | yes | Pseudo-alignment with Kallisto, followed by an iterative (EM) algorithm for transcript quantification |
| HLA-HD | Bowtie2 | yes | no | yes^*^ | Optimizes a score per allele pair which considers the overlap length between reads and exon sequences. |
| PHLAT | Bowtie2 | yes | yes | yes^*^ | Bayesian framework with likelihood calculated based on sequence consistency at SNP sites and phase consistency across adjacent SNP sites |
| Polysolver | Novoalign | no | yes | optional | Bayesian framework.  First determines the first allele, then identifies the second allele in a separate round with updated probabilities. |
| HLA-VBSeq | BWA-MEM | no | no | no | First optimizes read alignments using a Bayesian framework. Then HLA types are inferred based on depth of coverage per allele. Alleles that do not pass coverage threshold are filtered out. |
| seq2HLA | Bowtie | no | no | no | Reads associated with first found allele removed before determining second allele.  First genotyping at 2-digit level, before extended to 4-digit level. |
| Optitype | RazerS3 | yes | no | no | Uses ILP to find the set of MHC-I allele pairs that simultaneously explain the input data the best.  Does not consider rare alleles (not present in Allele Frequency Net) |
| xHLA | DIAMOND | hybrid | no | no | Identifies candidate alleles by applying Optitype’s ILP strategy on the PBR exons with subsequent iterative refinement steps. |
| HLAscan | BWA-MEM | no | no | no | Alleles discarded based on number of consecutive positions with no read aligned to it |
| HLAforest | Bowtie | no | yes | no | SMMQ scores are propagated throughout trees that represent all possible alignments per read. |
| HLAminer | BWA-backtrack | no | no | no | Supports assembling reads into longer contigs before comparing them to known allele sequences^†^. |

* only used for breaking-ties in case of ambiguities

† Only in Targeted Assembly of Shotgun Reads (HPTASR) mode, which was not evaluated in this paper

**Table S2.** Main algorithmic characteristics of the 13 selected HLA genotyping algorithms. Algorithms differ in how reads are aligned to the HLA allele reference sequences (column A*lignment method*) and how they subsequently score candidate alleles (column *Score function*). Commonly used variables of the score function are: base quality scores (column *PHRED score used*) and whether they use prior population frequencies (column *Prior population frequencies*). The score function can either be jointly optimized for allele pairs (column *Jointly optimized for allele pair*) or be defined on a single allele at once. The tools that have the value “hybrid” in this column combine both types of scoring functions. The column *tool-specific steps* describes additional particularities of the tools. More information is provided in *Additional file 3: Supplementary Note*.
