# Supplementary material for: Benchmark of tools for in silico prediction of MHC class I and class II genotypes from NGS data
Source: BMC Genomics. 2023 May 9;24:247. doi: 10.1186/s12864-023-09351-z (PMC10170851; doi:10.1186/s12864-023-09351-z)
Supplement: Supplementary file 3 — Supplementary Material 3 [file 12864_2023_9351_MOESM3_ESM.docx]

# Overview of HLA genotyping strategy used by 13 selected tools

## General principles

Reads (or assembled contigs) are aligned to a panel of reference HLA allele sequences (originating from the IPD-IMGT/HLA database). For this task the algorithms rely on various existing alignment tools (column *Alignment Method,* Table S2).

Then, for each HLA gene, an allele pair is selected by optimizing a certain score function. Algorithms differ in how this optimization problem is modeled exactly (e.g., Bayesian interference, Integer Linear Programming, or as a graph problem) and which variables are considered in the score function (column *Score function*, Table S2). Commonly used variables of the score function are: the consistency of the alignment of the input reads to the reference panel, base quality scores (column *PHRED score used*) and whether they use prior population frequencies (column *prior population frequencies*, Table S2).

This score function can either be defined in function of allele pairs for multiple genes at once (e.g., *Optitype*), in function of an allele pair per gene (e.g., *HLA*LA*) or in function of individual alleles separately per chromosome (e.g., *Polysolver*) (column *Jointly optimized for allele pair*, Table S2). In the first two cases, heterozygosity / homozygosity are implicitly modeled in the optimization problem. In the latter case, a separate step is typically needed to determine whether the call is homozygous or not based on numeric thresholds (e.g., for *HLA-VBSeq*).

## Tool-specific steps

The column *tool-specific* steps in Table S2 describes additional particularities of the tools:

### *HLA*LA*

*HLA*LA* starts with a “linear alignment” step where the input reads are aligned using *BWA‑MEM* to a modified reference genome (composed of GRCh38, the MHC haplotypes and IMGT genomic sequences). These alignments are then projected onto a population reference graph (PRG) and further optimized. Finally, the HLA genotype is inferred by maximizing a likelihood function [1].

### *Kourami*

*Kourami* is a graph-guided assembly tool. As a first step, reads are extracted from the BAM file and realigned to a reference panel (using *BWA‑MEM*). Similar as in *HLA*LA* these “linear alignments” are then projected onto a partial-order graph (POG) representing the known HLA alleles. During this projection step, the graph is modified to incorporate substitutions and indels that were identified during the alignment. This allows *Kourami* (as the only tool included in our benchmark) to discover new HLA alleles. Edges of the graph are weighted according to the read counts. Finally, the goal is to identify the best pair of alleles (paths in the graph) that maximizes the coverage and phasing support [2, 3].

### *arcasHLA*

First, pseudoalignment with *Kallisto* is performed to determine for each read which HLA transcripts it is compatible with. Based on this output, a built-in transcript quantification step is performed that aims to identify an attribution of reads to alleles which maximizes a likelihood function. This is performed using an iterative read re-allocation procedure (an expectation-maximization algorithm): at every step, reads are distributed to alleles with the highest abundance, while the alleles with the lowest abundances are removed from the list of candidates. When after convergence more than two alleles remain, the allele pair that explains the greatest proportion of reads is selected. Finally, either a homozygous or heterozygous call is produced based on the non-shared read counts between the top two alleles [4, 5].

### *HLA-HD*

First, reads are aligned to a database of reference HLA exon and intron sequences using *Bowtie2*. Reads are then assigned to candidate exons or introns based on certain filter criteria. A score is calculated per allele pair based on the number of reads that map to the corresponding sequences, considering the length of the overlap between input reads and exon sequences. The algorithm first calculates the score only based on exons in the peptide binding region and later extends to other exons. The allele pair that yields the maximum score is finally selected [6].

### *PHLAT*

*PHLAT* starts by aligning the input reads to the human reference genome extended with various HLA allele reference sequences using *Bowtie2*. Following the alignment, candidate alleles are pre-selected using multiple filtering steps based on mapped read counts. Pairs of candidate alleles are then scored using a Bayesian likelihood model which considers the sequence consistency at SNP sites and phase consistency across adjacent SNP sites. The allele pair that best explains the observed data is selected [7, 8].

### *Polysolver*

### Reads are first mapped to the reference allele sequences using *Novoalign*. *Polysolver* relies on a Bayesian classifier to select the alleles that most likely explain the observed reads. Its model incorporates the base qualities of aligned reads, the observed insert sizes and (optionally) ethnicity dependent prior probabilities. For each gene, the calls for both alleles are determined in separate steps. Once the first allele is identified, the probabilities are updated based on that information and the second allele for that locus is identified [9].

### *HLA-VBSeq*

Reads are aligned to the reference panel using *BWA‑MEM*. These alignments are then further optimized using a Bayesian framework. HLA types are subsequently called based on the expected number of reads that are mapped to each allele. A threshold on the depth of coverage is used to filter out candidate alleles. Either a heterozygous or homozygous call is outputted based on the depth of coverage of the top two ranked alleles [10].

### *seq2HLA*

*seq2HLA* uses a two-stage approach to accomplish HLA genotyping at 4-digit resolution: genotypes are first called at 2-digit resolution before further refining them in a separate round. The algorithm starts by aligning RNA-seq reads to the reference HLA panel with *Bowtie* (v1). Then, for each HLA gene the allele group with the greatest number of reads is determined and considered to be the winner of the first round. The second allele group for that gene is then determined by removing all reads associated with the winner of the first round and repeating the previous step. Either a heterozygous or homozygous call is outputted depending on the ratio between the number of reads mapping to the winner of the second round and the median number of reads mapped to alleles in the first round [11]. Finally, the allele calls at 2-digit resolution are further refined to the 4-digit resolution by considering the amount of reads aligned to alleles within the winning group at 2-digit resolution [12].

### *Optitype*

First, reads are mapped to a panel of reference HLA allele sequences, limited to the exons encoding the peptide binding region of MHC-I (exon 2 and 3) and the flanking introns. Partial HLA sequencing information in the reference panel was reconstructed using phylogenetic information. *Optitype* then models the scoring of HLA alleles as an Integer Linear Programming (ILP) problem that aims to find the set of MHC-I allele pairs (for all major and minor MHC-I genes) that simultaneously explain the input data the best [13].

### *xHLA*

*xHLA* maps the sequencing reads to the HLA reference sequences using the *Diamond* aligner and subsequently identifies candidate alleles by applying *Optitype*’s ILP strategy using only the exons encoding the peptide binding region (for both MHC-I and MHC-II genes). This set of candidate alleles is subsequently extended to sets of alleles that explain the alignments nearly as well and then further refined using an iterative procedure. This strategy allows HLA genotyping at a finer resolution than *Optitype*. When finally two alleles remain, an additional check is performed to determine whether a homozygous or heterozygous call should be outputted by comparing the amount of reads supporting both alleles [14].

### *HLAscan*

After aligning reads to the reference sequences (*BWA‑MEM*), *HLAscan* selects candidate alleles based on a score function that represents the distribution of aligned reads in the region of interest. Alleles are discarded based on the number of consecutive positions in the mapped HLA sequence with no read aligned to it. Out of the remaining alleles, the resulting allele pairs for each gene are determined based on which alleles have the highest read count and a check for heterozygosity [15].

### *HLAforest*

Reads are mapped to the HLA reference allele sequences using *Bowtie* (v1). For each read a tree is constructed that represents all possible mappings for that read. The first level of the tree represents the different HLA genes, each subsequent level represents a different field of the HLA nomenclature. Sum of mismatch qualities (SMMQs, based on the PHRED qualities at mismatches between the read and reference sequence) are assigned to the leaf nodes of the tree. This score is than propagated upwards the tree where the probability value assigned to a parent node is the maximum probability of its children. These probability values are then converted into *weights*, which are distributed downwards through the tree. The final allele pair is then selected via an iterative tree pruning algorithm [16].

### *HLAminer*

*HLAminer* supports both a *de novo* assembly-based (HPTASR) and an alignment-based (HPRA) pipeline. In the de novo assembly-based pipeline (not evaluated in this benchmark) reads are first assembled into larger contigs and are subsequently aligned to the panel of reference HLA sequences (using *BLAST*). In the alignment-based pipeline the reads are directly aligned to the reference allele sequences using *BWA*. Alleles are scored based on the contig length, depth of coverage and similarity to reference sequences of all contigs that align to it [17]. *HLAminer* does not incorporate a method to impute heterozygosity / homozygosity.

# References

1. Dilthey AT, Mentzer AJ, Carapito R, Cutland C, Cereb N, Madhi SA, et al. HLA*LA—HLA typing from linearly projected graph alignments. Bioinformatics. 2019;35:4394–6.

2. Lee H, Kingsford C. Kourami: Graph-guided assembly for novel human leukocyte antigen allele discovery. Genome Biol. 2018;19:1–16.

3. Lee H, Kingsford C. Accurate assembly and typing of HLA using a graph-guided assembler kourami. Methods in Molecular Biology. 2018;1802:235–47.

4. Orenbuch R, Filip I, Rabadan R. HLA typing from RNA sequencing and applications to cancer. Methods in Molecular Biology. 2020;2120:71–92.

5. Orenbuch R, Filip I, Comito D, Shaman J, Pe’Er I, Rabadan R. arcasHLA: high-resolution HLA typing from RNAseq. Bioinformatics. 2020;36:33–40.

6. Kawaguchi S, Higasa K, Shimizu M, Yamada R, Matsuda F. HLA-HD: An accurate HLA typing algorithm for next-generation sequencing data. Hum Mutat. 2017;38:788–97.

7. Bai Y, Wang D, Fury W. PHLAT: Inference of high-resolution HLA types from RNA and whole exome sequencing. Methods in Molecular Biology. 2018;1802:193–201.

8. Bai Y, Ni M, Cooper B, Wei Y, Fury W. Inference of high resolution HLA types using genome-wide RNA or DNA sequencing reads. BMC Genomics. 2014;15:1–16.

9. Shukla SA, Rooney MS, Rajasagi M, Tiao G, Dixon PM, Lawrence MS, et al. Comprehensive analysis of cancer-associated somatic mutations in class I HLA genes. Nat Biotechnol. 2015;33:1152.

10. Nariai N, Kojima K, Saito S, Mimori T, Sato Y, Kawai Y, et al. HLA-VBSeq: Accurate HLA typing at full resolution from whole-genome sequencing data. BMC Genomics. 2015;16:1–6.

11. Boegel S, Löwer M, Schäfer M, Bukur T, de Graaf J, Boisguérin V, et al. HLA typing from RNA-Seq sequence reads. Genome Med. 2012;4:1–12.

12. Boegel S, Löwer M, Bukur T, Sahin U, Castle JC. A catalog of HLA type, HLA expression, and neoepitope candidates in human cancer cell lines. Oncoimmunology. 2014;3.

13. Szolek A, Schubert B, Mohr C, Sturm M, Feldhahn M, Kohlbacher O. OptiType: precision HLA typing from next-generation sequencing data. Bioinformatics. 2014;30:3310–6.

14. Xie C, Yeo ZX, Wong M, Piper J, Long T, Kirkness EF, et al. Fast and accurate HLA typing from short-read next-generation sequence data with xHLA. Proceedings of the National Academy of Sciences. 2017;114:8059–64.

15. Ka S, Lee S, Hong J, Cho Y, Sung J, Kim HN, et al. HLAscan: Genotyping of the HLA region using next-generation sequencing data. BMC Bioinformatics. 2017;18:1–11.

16. Kim HJ, Pourmand N. HLA Haplotyping from RNA-seq Data Using Hierarchical Read Weighting. PLoS One. 2013;8:e67885.

17. Warren RL, Choe G, Freeman DJ, Castellarin M, Munro S, Moore R, et al. Derivation of HLA types from shotgun sequence datasets. Genome Med. 2012;4:1–8.
