# Supplementary material for: Benchmark of tools for in silico prediction of MHC class I and class II genotypes from NGS data
Source: BMC Genomics. 2023 May 9;24:247. doi: 10.1186/s12864-023-09351-z (PMC10170851; doi:10.1186/s12864-023-09351-z)
Supplement: Supplementary file 4 — Supplementary Material 4 [file 12864_2023_9351_MOESM4_ESM.pdf]

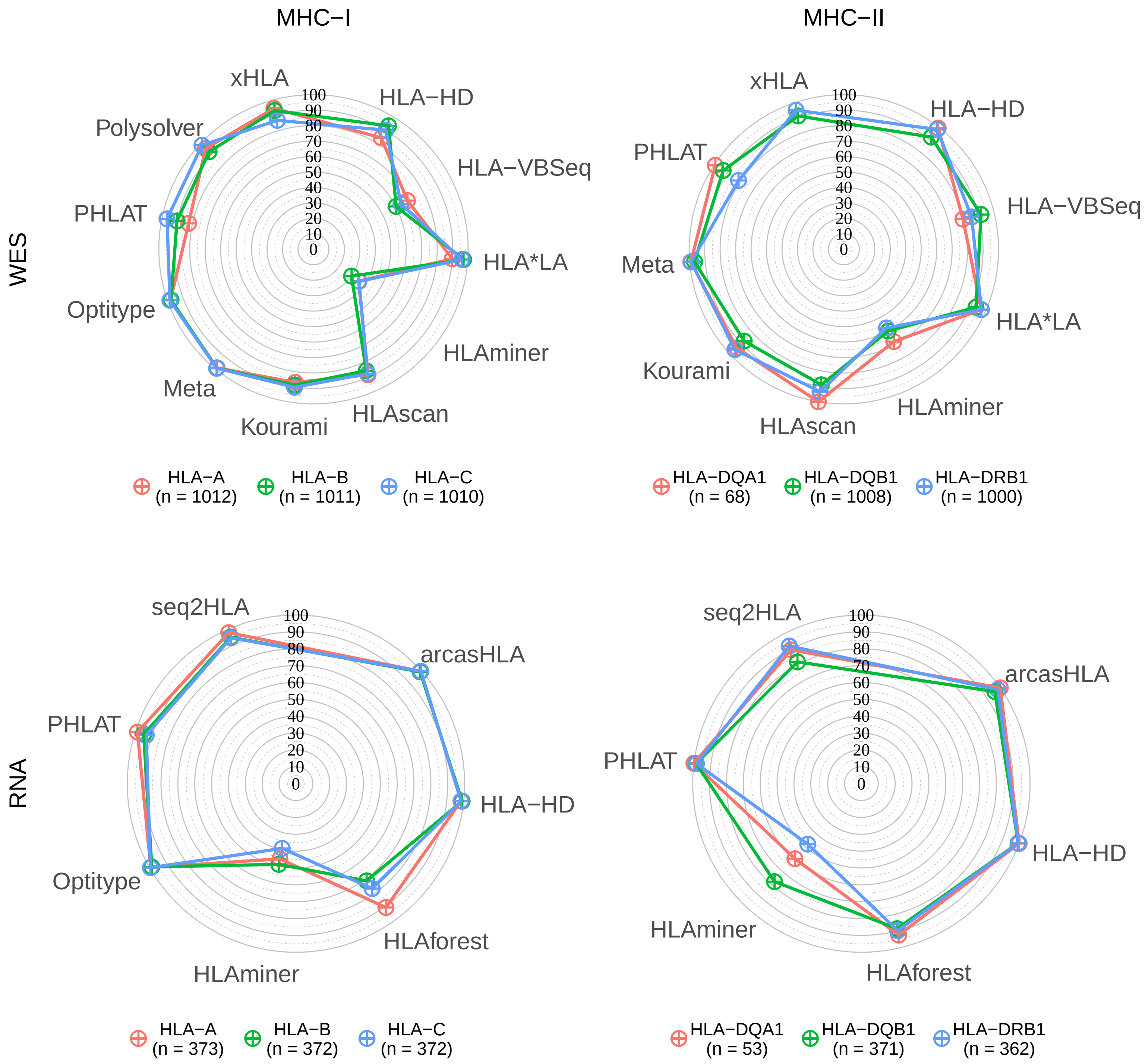

Figure S1: Fraction of correct allele predictions (1000 genomes)
Radar plots depicting the fraction of correct allele predictions relative to the total number of alleles for which the algorithm was able to make a prediction on the 1000 genomes dataset. Coloured lines represent different genes, as indicated in the legend below the plots. Corners of the radar plots correspond to the tools that were evaluated for that data type. The Meta tools correspond to the 4-tools metaclassifiers.

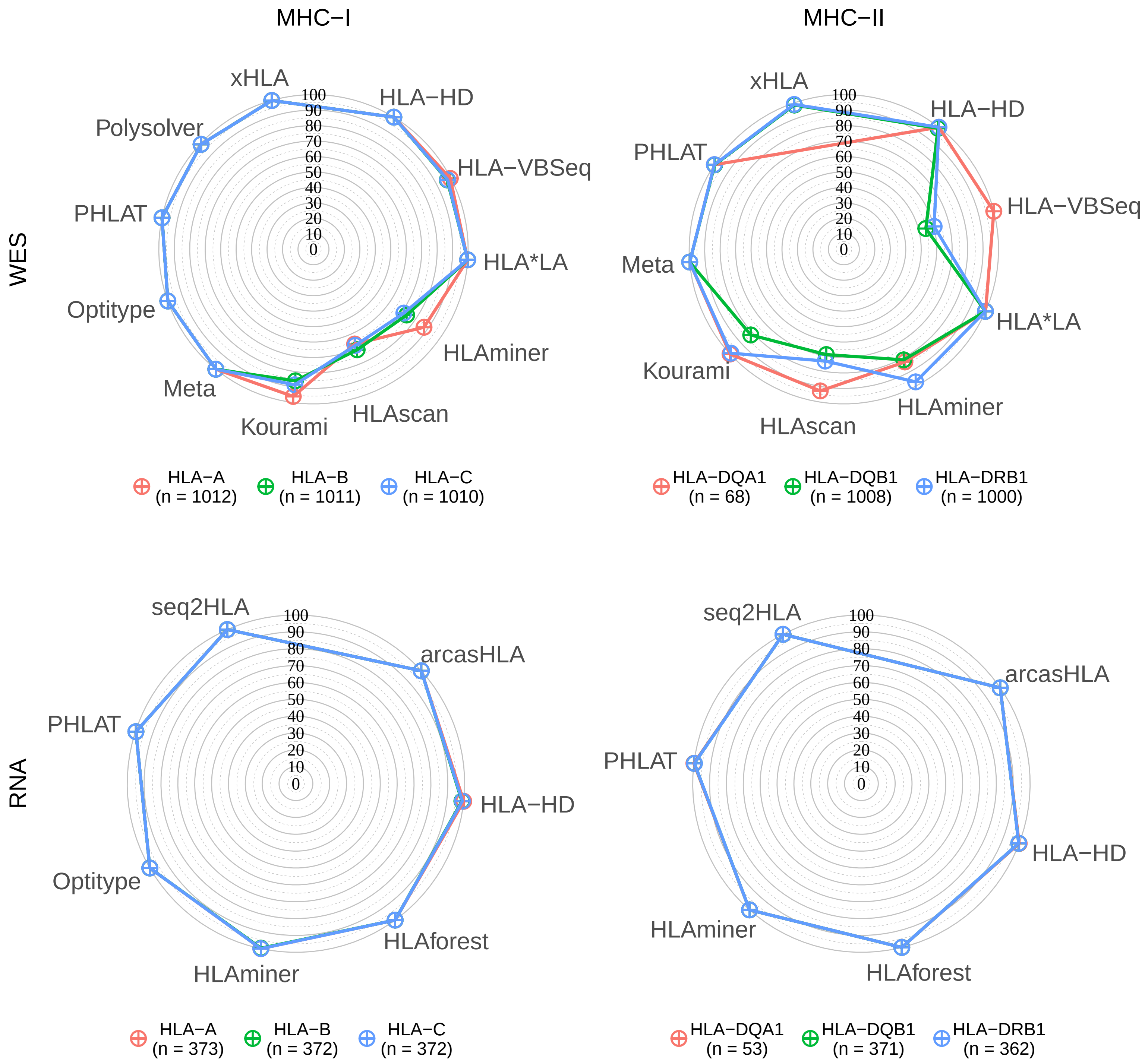

Figure S2: Fraction of successful allele predictions (1000 genomes)
Radar plots depicting the fraction of alleles for which the tool was able to make a prediction on the 1000 genomes dataset. Coloured lines represent different genes, as indicated in the legend below the plots. Corners of the radar plots correspond to the tools that were evaluated for that data type. The Meta tools correspond to the 4-tools metaclassifiers.

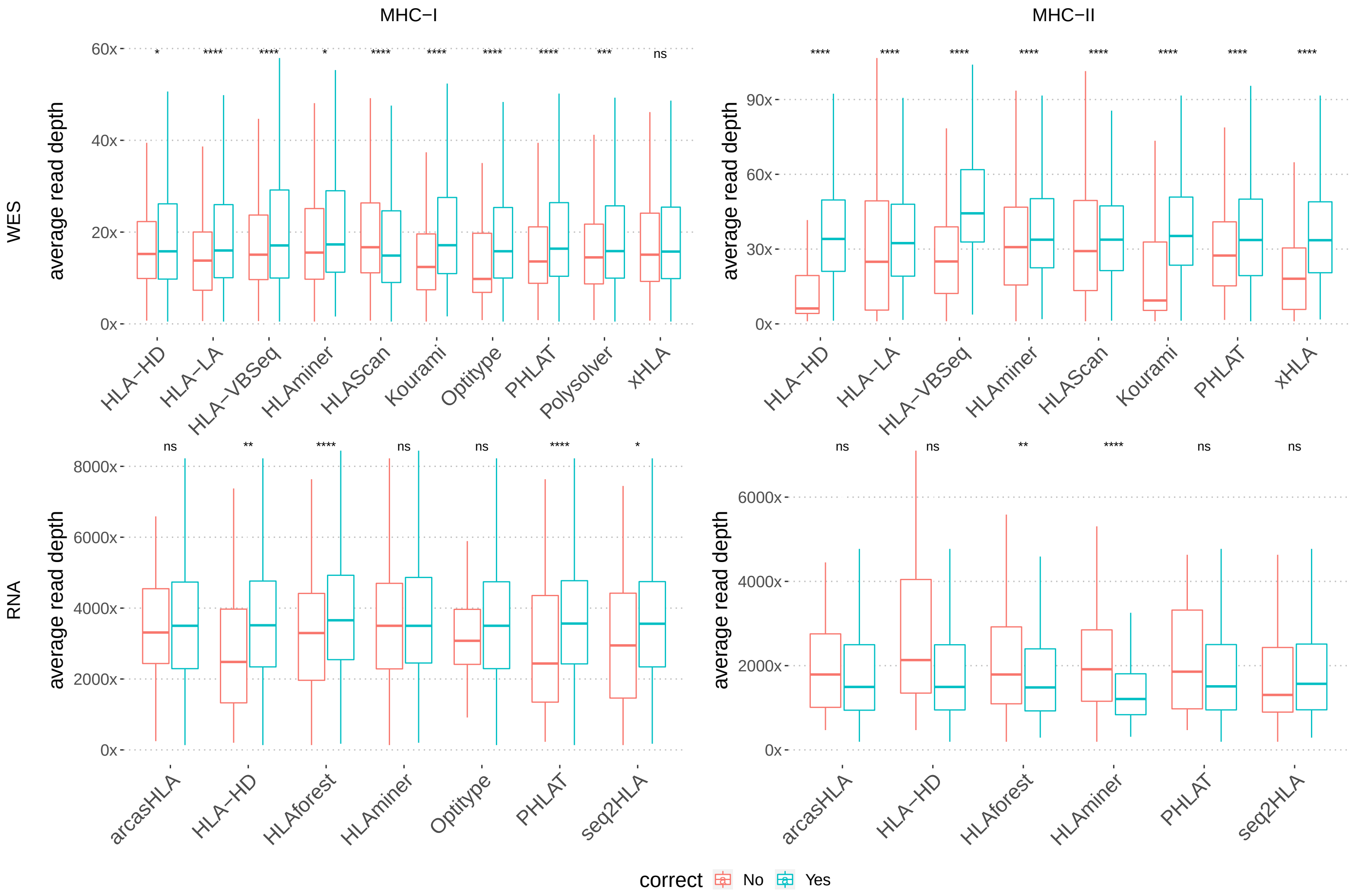

Figure S3: Comparison between the average HLA read depth for correct and incorrect predictions Boxplot comparing the average HLA read depth in samples and genes that were either correctly (cyan) or incorrectly (red) predicted. The y-axis indicates the coverage in these exons. The x-axis indicates the different tools.

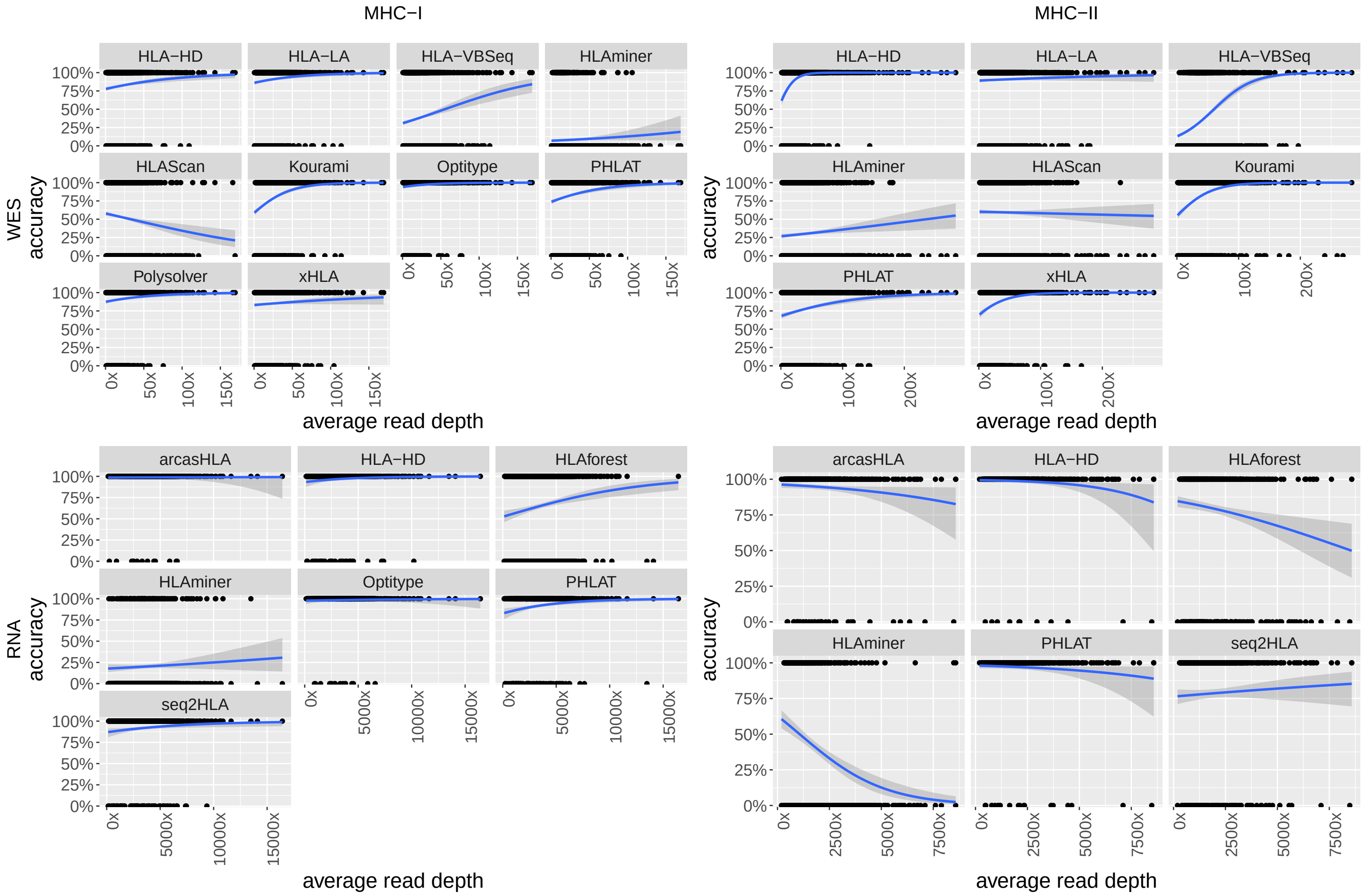

Figure S4: Logistic regression between average HLA read depth and the accuracy of the allele predictions
Logistic regression model that relates the average HLA read depth with the correctness of the allele pair prediction. The x-axis indicates the coverage. The y-axis indicates the probability that a prediction is correct for a sample with the corresponding coverage.

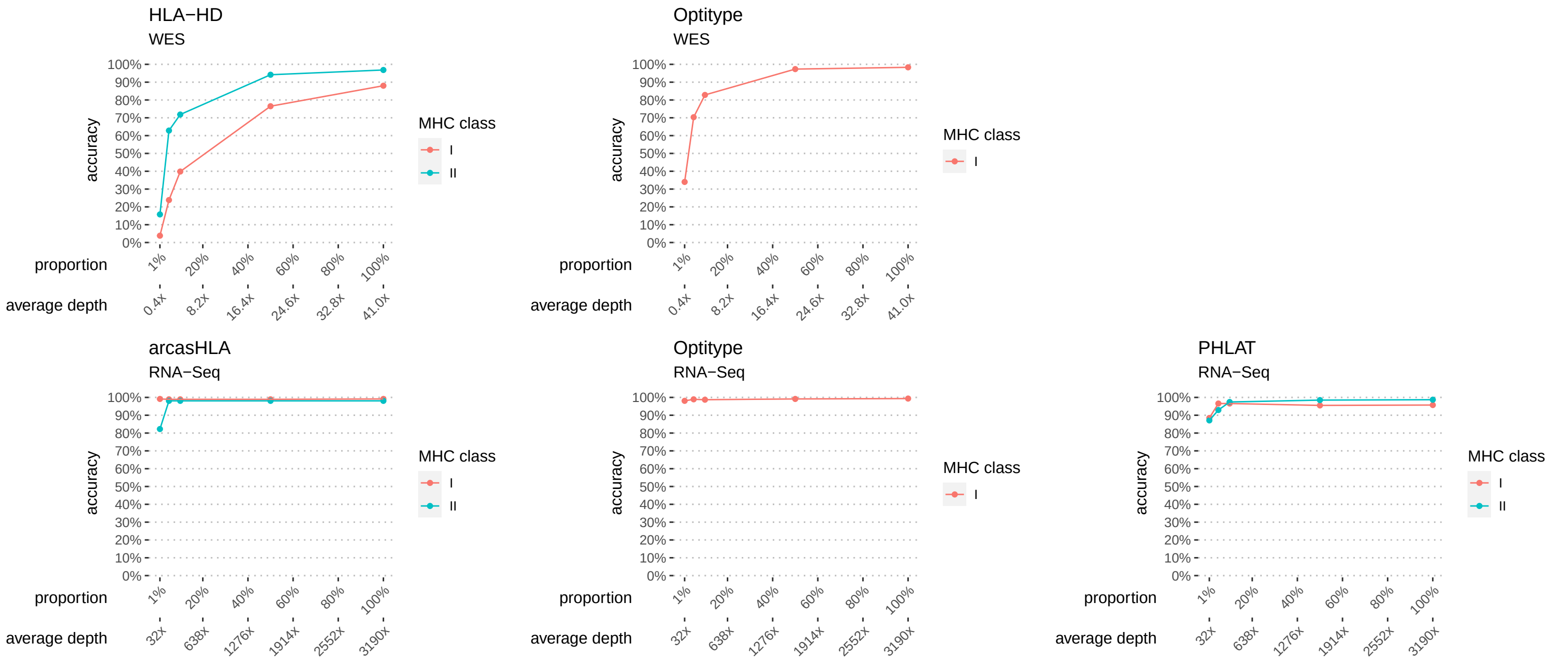

Figure S5: Accuracy of HLA allele predictions in subsampled sequencing files for the recommended tools
Scatter plot that displays for 100 randomly selected WES and 100 randomly selected RNA sequencing files which accuracy was be obtained when a given proportion of the reads was retained. The line type indicates the average accuracy of the allele predictions for a certain MHC class. The colour of the lines indicates the data type (red for WES and cyan for RNA-Seq).

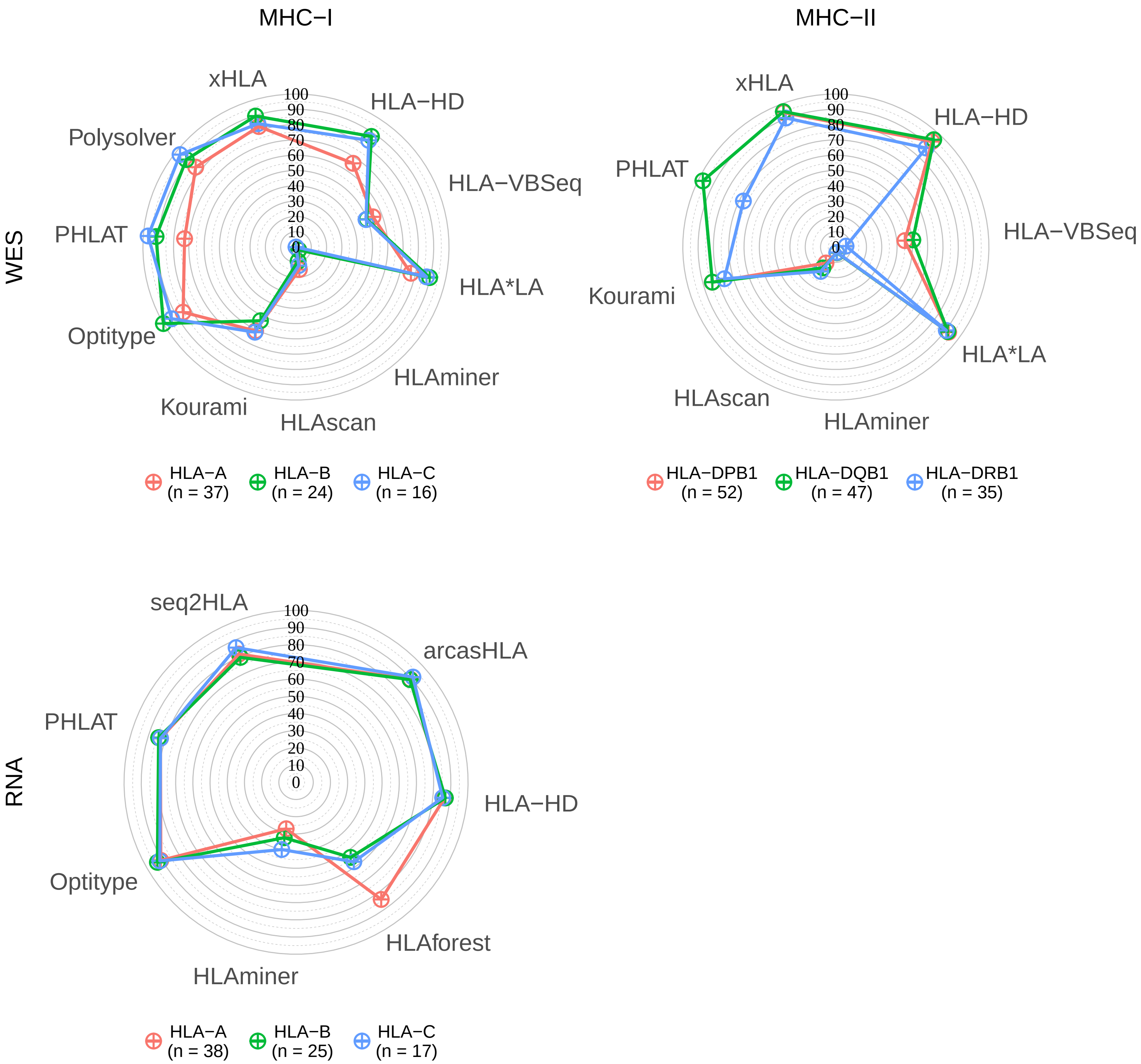

Figure S6: HLA allele prediction accuracies on NCI-60 cell lines
Radar plots of HLA allele prediction accuracies on data from NCI-60 cell lines. Coloured lines represent different genes, as indicated in the legend below the plots. Corners of the radar plots correspond to the tools that were evaluated for that data type.

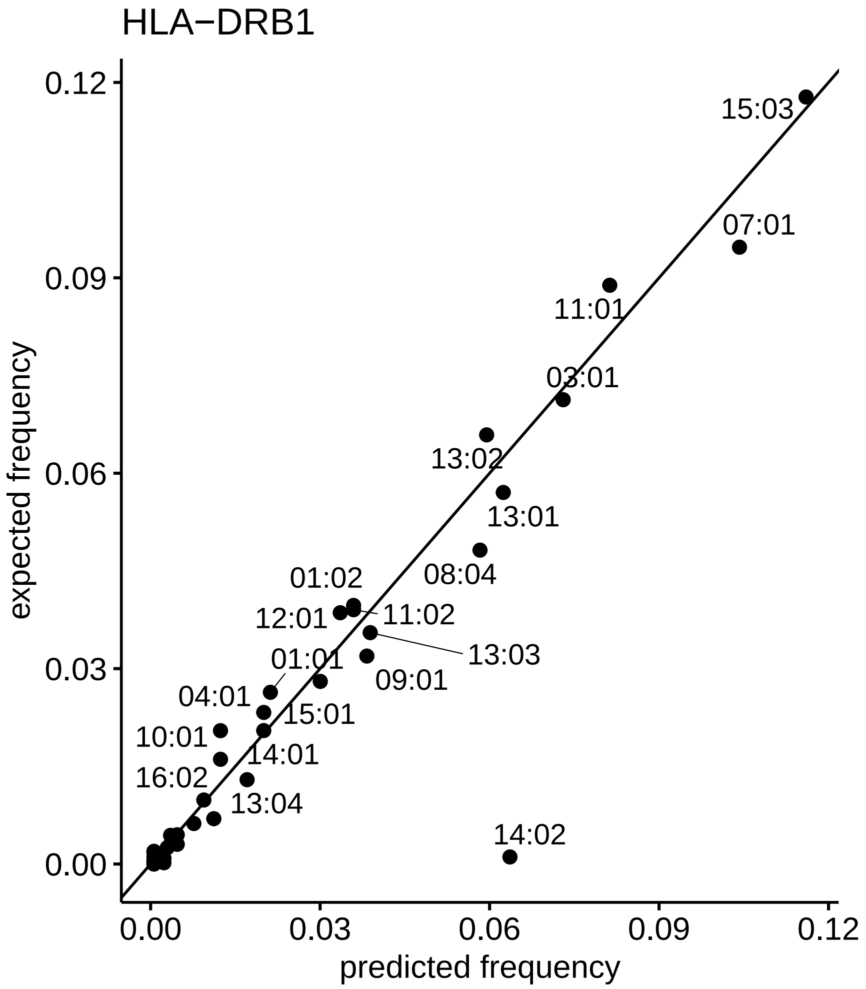

Figure S7: Expected frequency of HLA-DRB1 alleles in an African American population vs frequencies predicted by arcasHLA
Scatter plot that compares the allele frequency as predicted by arcasHLA (x-axis) with the expected allele frequencies based on data from Allele Frequency Net (y-axis).

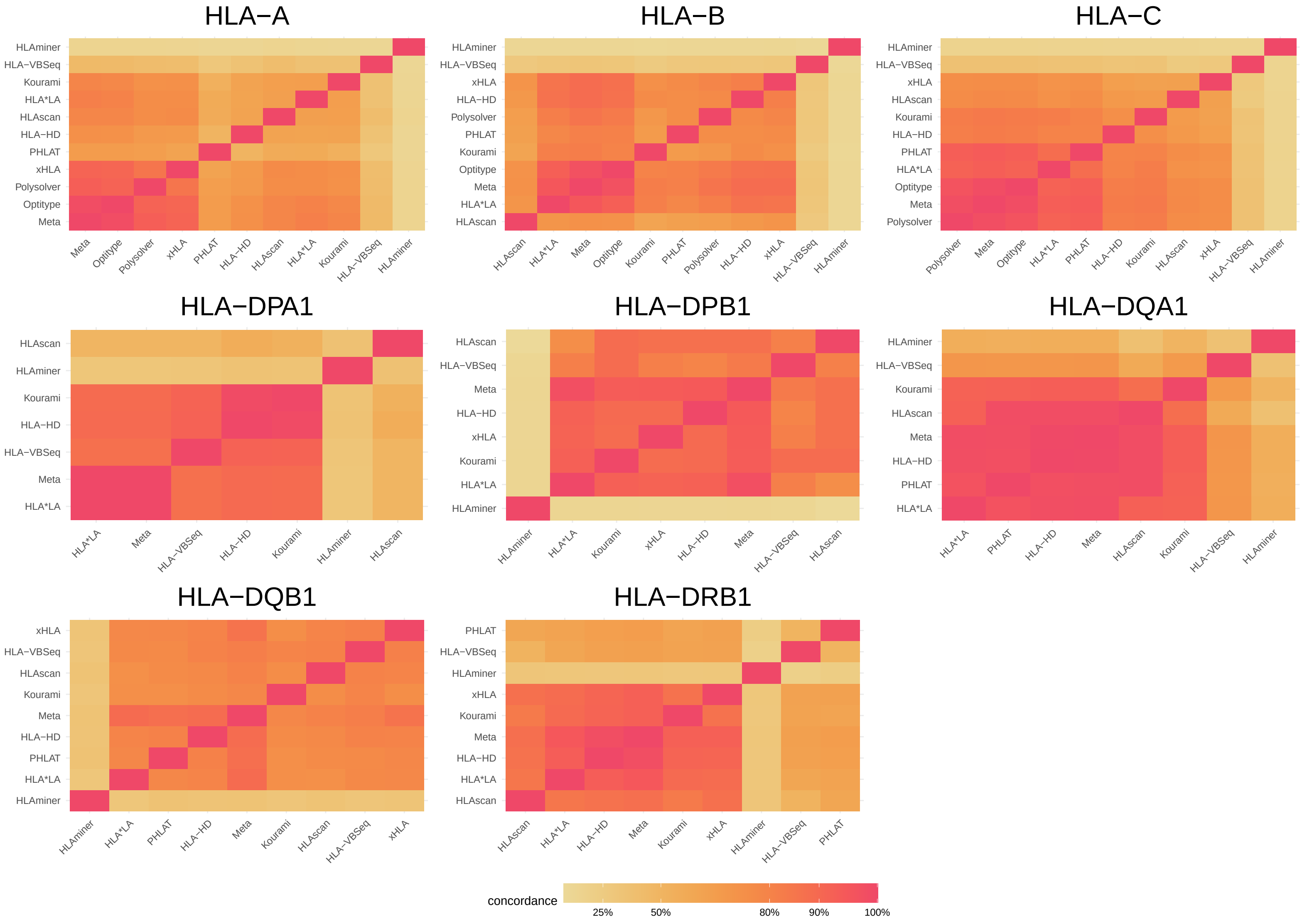

Figure S8: Concordance of HLA calls between each pair of tools on DNA data (1000 genomes) Heatmaps representing the concordance of the HLA calls between each pair of tools, applied on the 1000 genomes DNA data. Hierarchical clustering was applied on the tools. The Meta tool corresponds to the 4-tool consensus metaclassifier.

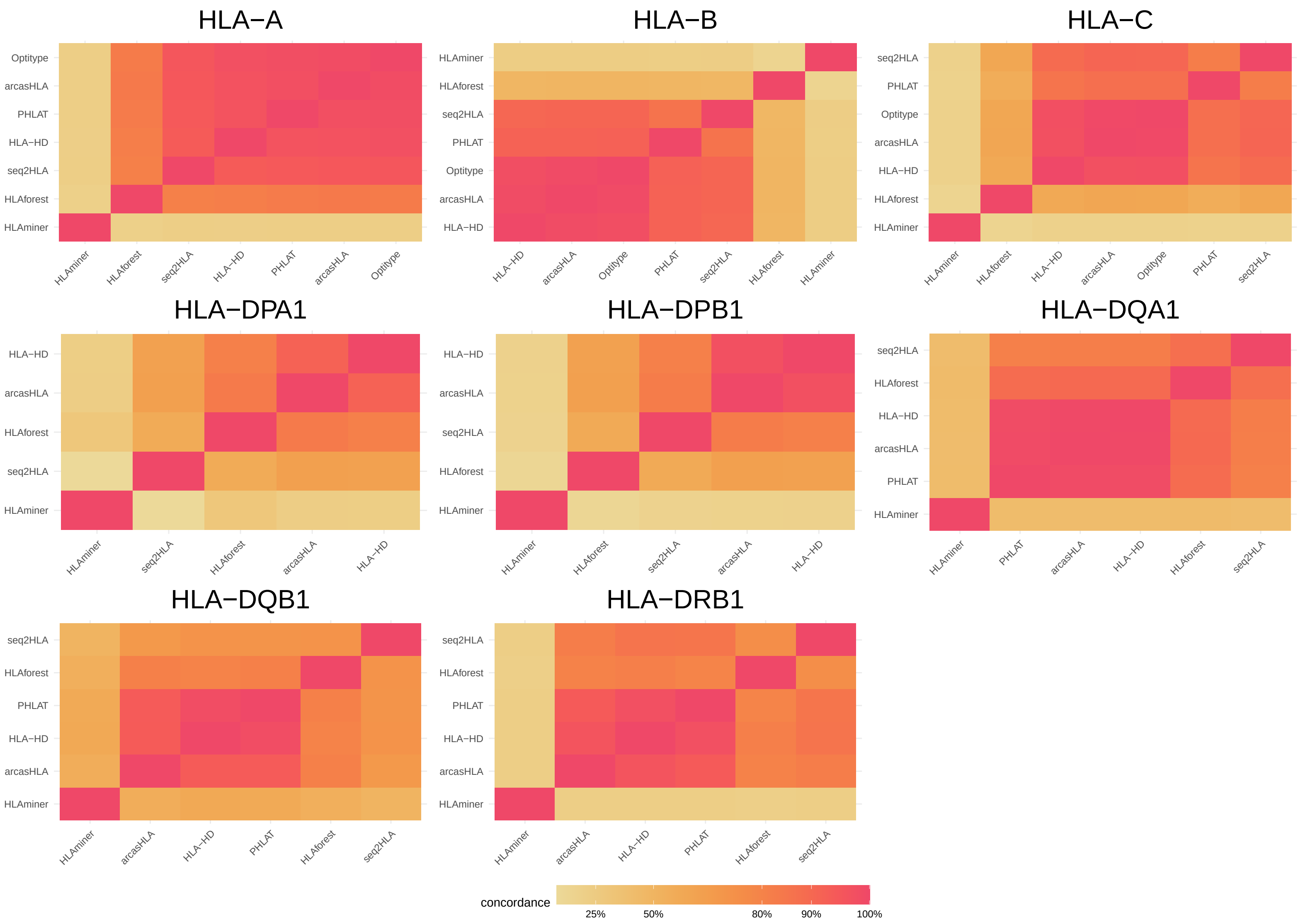

Figure S9: Concordance of HLA calls between each pair of tools on RNA data (1000 genomes)
Heatmaps representing the concordance of the HLA calls between each pair of tools, applied on the 1000 genomes RNA data. Hierarchical clustering was applied on the tools.

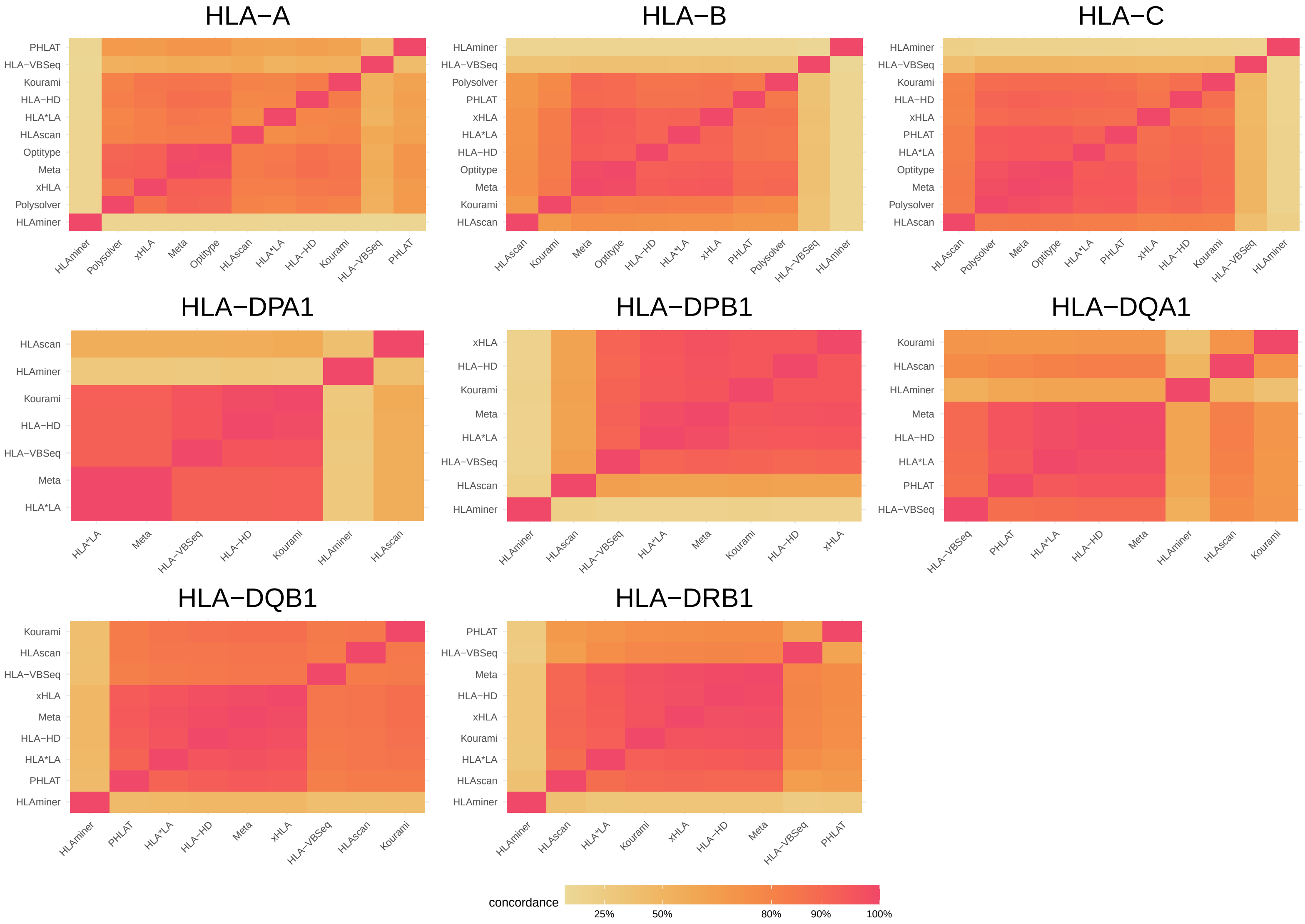

Figure S10: Concordance of HLA calls between each pair of tools on DNA data (TCGA) Heatmaps representing the concordance of the HLA calls between each pair of tools, applied on the TCGA DNA data. Hierarchical clustering was applied on the tools. The Meta tool corresponds to the 4-tool consensus metaclassifier.

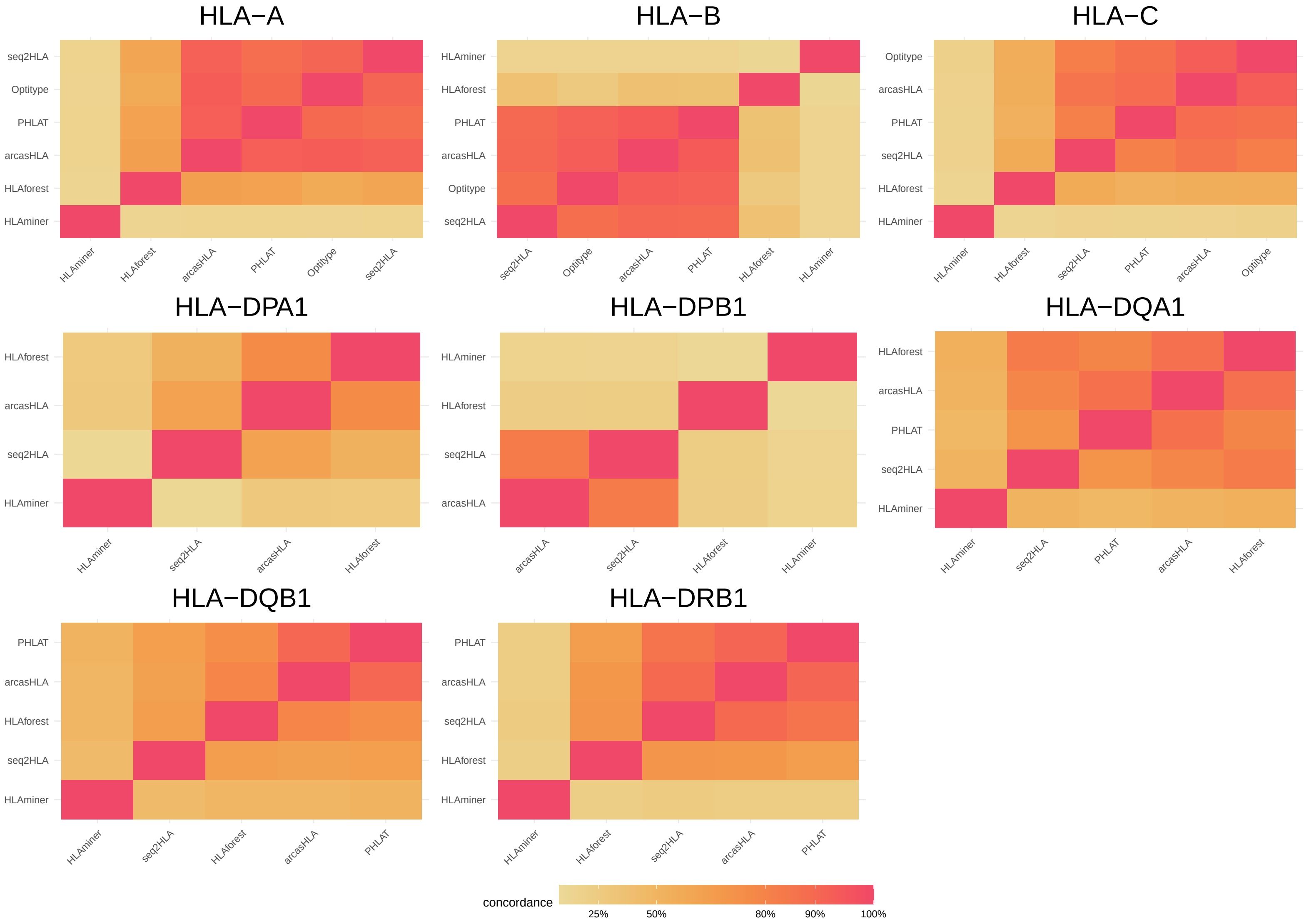

Figure S11: Concordance of HLA calls between each pair of tools on RNA data (TCGA)
Heatmaps representing the concordance of the HLA calls between each pair of tools, applied on the TCGA RNA data. Hierarchical clustering was applied on the tools.

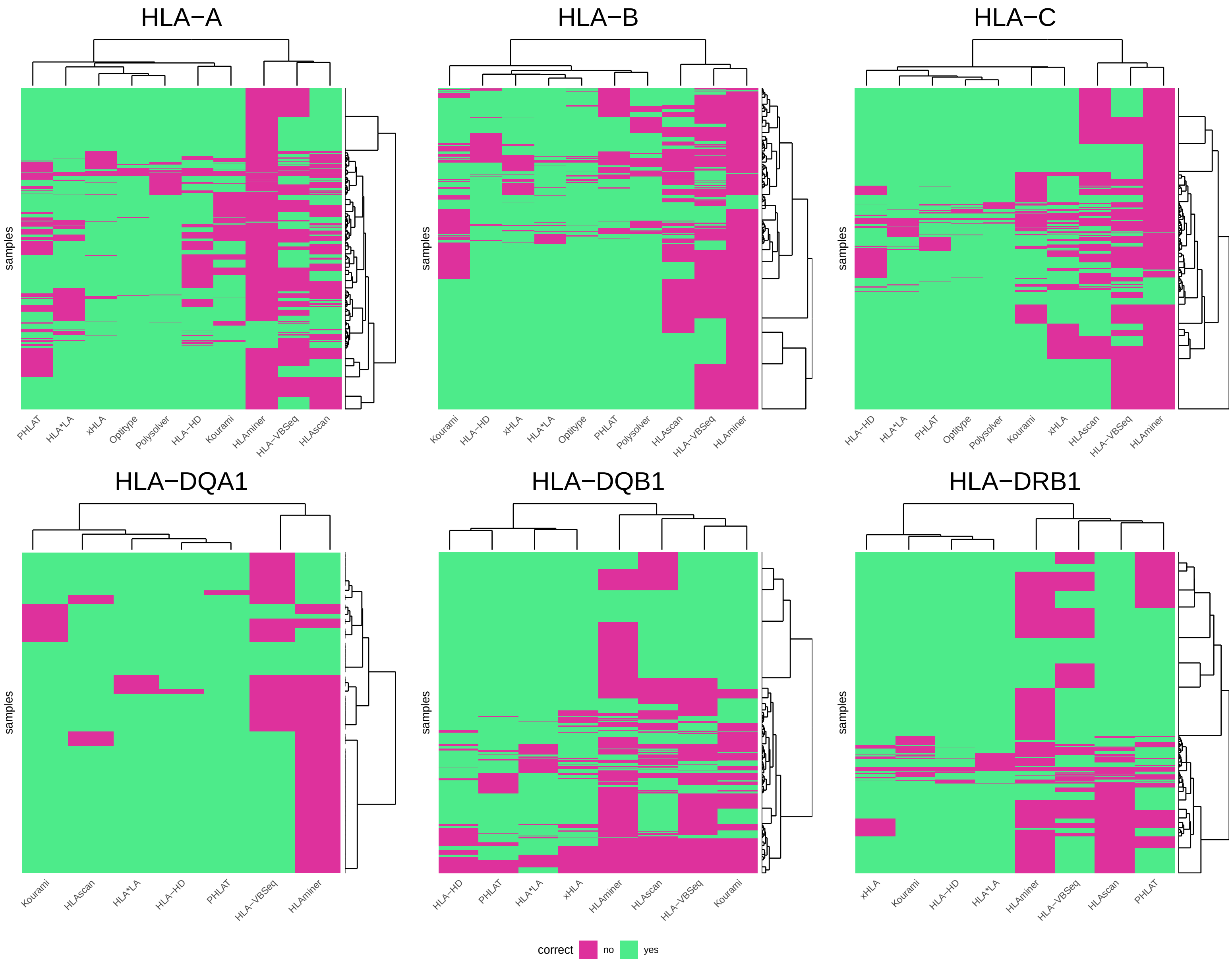

Figure S12: Correctness of predictions on DNA data
Heatmap indicating correctness of predictions on DNA data for each sample (rows) and tool (columns). Hierarchical clustering was applied on tools and samples. Dendrogram for the tools is shown on top of the plots. Dendrogram for the samples is shown right of the plots.

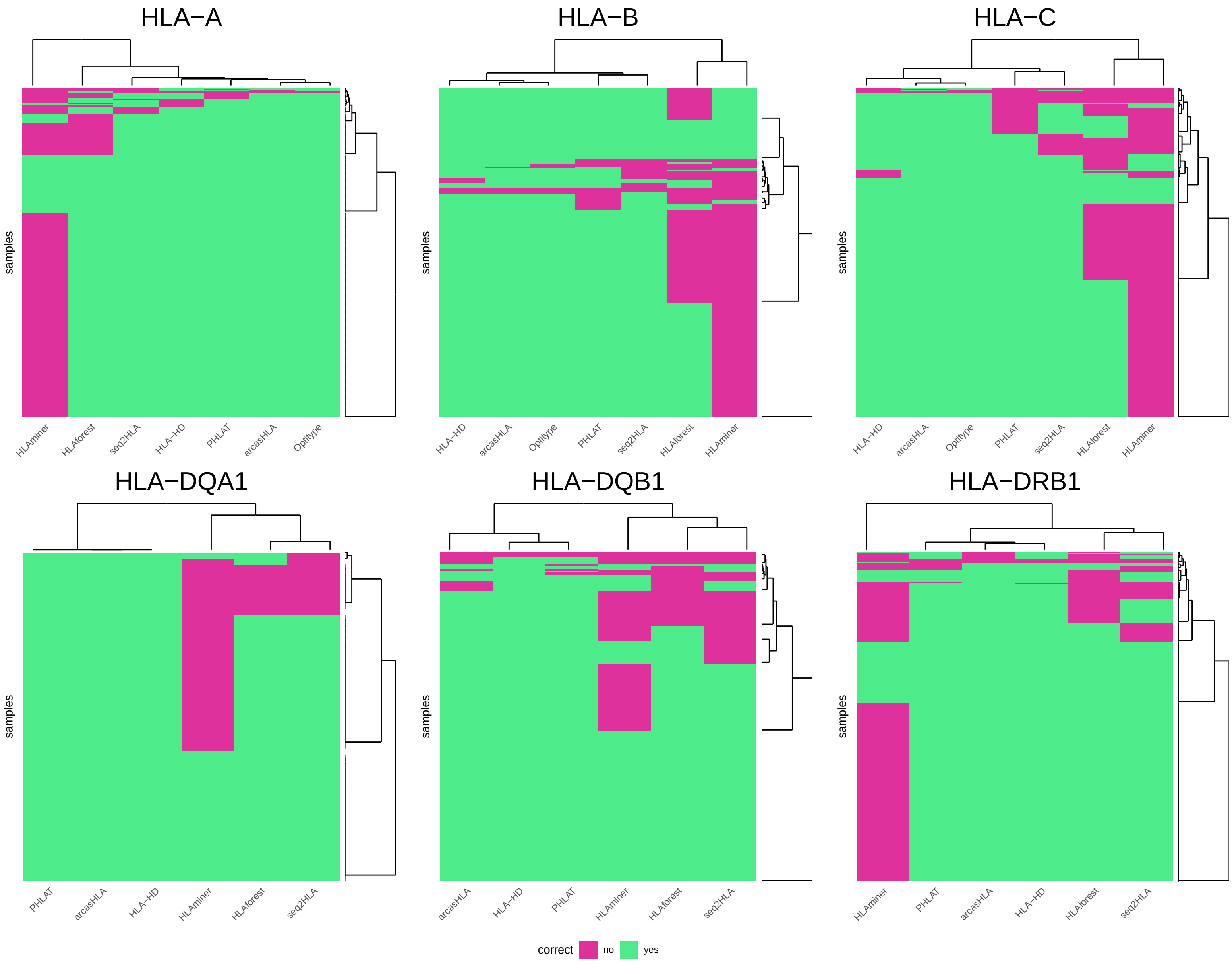

Figure S13: Correctness of predictions on RNA data
Heatmap indicating correctness of predictions on RNA data for each sample (rows) and tool (columns). Hierarchical clustering was applied on tools and samples. Dendrogram for the tools is shown on top of the plots. Dendrogram for the samples is shown right of the plots.

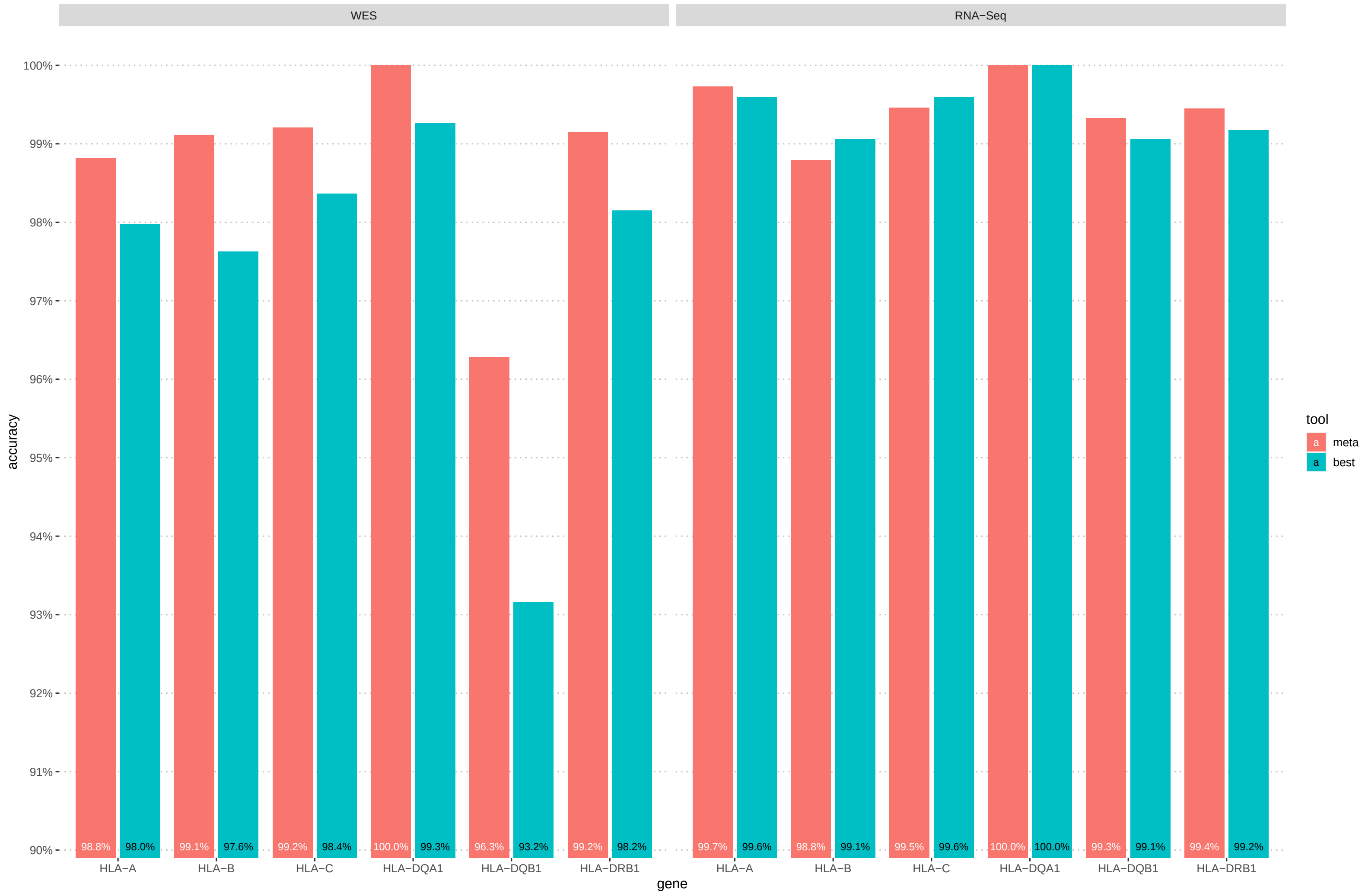

Figure S14: Comparison of accuracies of all-tool metaclassifier with best performing individual tool per gene

Barplots comparing the accuracy of the best tool for each gene and data type to the accuracy of a classifier that chooses an HLA genotype from the output of all tools that support that data type and gene based on a majority voting rule. Bars in a red correspond to the accuracies of the voting classifier. Bars in blue correspond to the accuracies of the best individual tool for that gene.
